# MUC2 shifts the metabolic profiles of commensal bacteria in defined microbial communities

**DOI:** 10.20517/mrr.2025.91

**Published:** 2026-01-21

**Authors:** Erin Chard, Adelaide E. Horvath, Makenna Grozis, Renata Rocha do Nascimento, Paul R. S. Baker, Santosh Kapil Kumar Gorti, Robert Proos, Thomas D. Horvath, Melinda A. Engevik

**Affiliations:** ^1^Department of Regenerative Medicine & Cell Biology, Medical University of South Carolina, Charleston, SC 29425, USA.; ^2^Department of Biology & Biochemistry, University of Houston, Houston, TX 77204, USA.; ^3^Department of Mathematics, University of Houston, Houston, TX 77204, USA.; ^4^Department of Biology, Elon University, Elon, NC 27244, USA.; ^5^Metabolomics & Lipidomics Applications, SCIEX, Marlborough, MA 01752, USA.; ^6^Department of Pathology & Immunology, Baylor College of Medicine, Houston, TX 77030, USA.; ^7^Department of Pathology, Texas Children’s Hospital, Houston, TX 77030, USA.; ^8^Department of Pharmacy Practice & Translational Research, College of Pharmacy, University of Houston, Houston, TX 77030, USA.; ^9^Department of Pharmacology & Immunology, Medical University of South Carolina, Charleston, SC 29425, USA.

**Keywords:** Bacteria, short-chain fatty acids, amino acids, mucus, metabolism

## Abstract

**Background:** The intestinal mucus layer is comprised of heavily glycosylated mucins, including mucin 2 (MUC2), that serve as a nutrient source for certain bacterial members of the gut microbiota. Only a subset of gut commensals encode the glycoside hydrolases required to degrade mucin glycans. However, mucin-degrading microbes can release glycans and generate compounds that can cross-fed non-mucin degrading microbes, creating complex microbial networks. While pairwise studies have shown that mucin degradation drives cross-feeding and metabolite exchange, the broader impact of mucins on community structure and metabolic output remains poorly understood.

**Objective:** In this study, we sought to identify how a defined microbial consortium of human commensals with varied mucin-degrading capacities responds to MUC2 to shape community composition and metabolic output.

**Methods: **A defined consortium of human gut commensals with varied mucin-degrading capacities was cultivated in anaerobic bioreactors in the presence or absence of porcine MUC2. Community composition was assessed, and extracellular metabolites were quantified using targeted and untargeted metabolomic profiling.

**Results: **MUC2 supplementation significantly altered community structure, promoting the expansion of *Akkermansia muciniphila* while reducing *Prevotella*. MUC2 also reshaped microbial metabolism, decreasing acetate levels while increasing propionate, butyrate, and formate. In addition, MUC2 supplementation altered amino acid utilization and vitamin metabolism and reduced several neuroactive compounds, including glutamate, γ-aminobutyric acid (GABA), and anthranilic acid, while increasing tryptamine levels.

**Conclusion: **These findings demonstrate that mucins exert broad effects on microbial community structure and metabolic output. Collectively, this work highlights the central role of bacterial cross-feeding in shaping gut ecosystem function.

## INTRODUCTION

The human gastrointestinal tract is lined with a dense mucus layer that plays a pivotal role in maintaining host-microbe homeostasis^[1-3]^. In the intestine, this mucus barrier is largely composed of mucin proteins, such as Mucin 2 (MUC2), which are extensively modified by O-linked glycosylation^[4-10]^. At serine and threonine residues on MUC2 proteins, O-linked glycans are formed by the addition of N-acetyl-galactosamine. Then, other core structures such as α- and β-linked N-acetyl-glucosamine, N-acetyl-galactosamine, and galactose can be added to the N-acetyl-glucosamine to create a variety of core structures. These cores are further elongated and modified with terminal sugars such as α-linked fucose, sialic acid, and sulfate groups, resulting in the characteristic bottle-brush architecture of mucus^[11]^. These mucin glycans are essential for generating a functionally mature mucus layer that retains water and maintains barrier function^[9,12-15]^. While historically considered a passive barrier, it is now clear that mucus is an active participant in shaping the intestinal ecosystem. In addition to protecting epithelial surfaces, mucins also serve as a niche and nutrient source for gut microbes^[16-24]^. The degradation of mucin glycans by specialized bacteria allows them to persist in nutrient-limited conditions, particularly in the distal colon, where dietary substrates are depleted. 

Only a subset of bacteria within the gut microbiota possess the enzymes to extensively deconstruct the complex O-glycan structures of mucins^[25,26]^. Notably, species such as *Akkermansia muciniphila* (*A. muciniphila*) and several members of the *Bacteroides* and *Ruminococcus* genus are considered keystone mucin degraders due to their extensive mucolytic capabilities^[25,27-34]^. These bacteria are equipped with an arsenal of glycan-degrading enzymes - also known as glycoside hydrolases (GH) - that enable them to liberate sugars from the mucin scaffold. In contrast, many commensals such as *Lactobacillus* and *Streptococcus* have a limited capacity to degrade mucins and instead rely on alternative nutrient sources^[25]^. This enzymatic asymmetry within the gut microbiota creates a metabolic hierarchy, where mucin degraders potentially support the growth of non-degraders through cross-feeding of liberated sugars or metabolites.

Bacterial cross-feeding, the process by which one microbial species uses metabolic byproducts generated by another, is increasingly recognized as a key mechanism governing community structure and function^[35-37]^. In the context of mucin degradation, released monosaccharides and amino acids can serve as substrates for neighboring microbes^[37,38]^. Additionally, the metabolic byproducts generated by mucin-degraders such as acetate and propionate can also serve as a substrate for non-degraders to generate compounds such as butyrate. Previous studies have demonstrated that mucin degradation by *A. muciniphila* results in byproducts such as acetate, propionate, succinate, and 1,2-propanediol which can cross-feed other gut bacteria in co-cultures *in vitro* and lead to the production of butyrate^[39-44]^. Similarly, mucin-degrading *Bifidobacterium bifidum* has also been shown *in vitro* to degrade mucins and cross-feed *Eubacterium hallii*, which in turn elevated the levels of butyrate^[45]^. Beyond short-chain fatty acids, the full spectrum of downstream metabolites generated through mucin-mediated cross-feeding remains poorly characterized. Furthermore, most existing studies have focused on pairwise interactions, which have provided mechanistic insights into bacterial cross-feeding, but may not represent the complex dynamics of the gut. 

To systematically address these gaps, we engineered a defined microbial consortium composed of human gut commensals with varied mucin-degrading capabilities. The designer community included robust mucin degraders (*Akkermansia*, *Bacteroides*), moderate degraders (*Blautia*, *Bifidobacterium*, *Enterococcus*, *Prevotella*), and non-degraders (*Lactobacillus*, *Lactococcus*, *Streptococcus*, *Clostrium*, *Escherichia*). By cultivating this community in anaerobic bioreactors and supplying exogenous MUC2, we sought to define how mucin substrates reshape bacterial composition and metabolite output in a controlled setting. Here, we demonstrate that the addition of porcine MUC2 significantly altered the microbial community structure, favoring expansion of mucin degrading specialists such as *A. muciniphila* and reducing the abundance of other taxa such as *Prevotella*. Importantly, this shift was accompanied by substantial changes in the metabolic landscape, including increased levels of formate, propionate, and butyrate, and a decrease in acetate and valerate. We also found that bacterial communities supplemented with MUC2 had reduced levels of glutamate (Glu), γ-aminobutyric acid (GABA), and anthranilic acid and elevated levels of tryptamine compared to control communities. These findings highlight the functional consequences of mucus degradation and underscore the importance of bacterial cross-feeding in shaping the gut ecosystem. 

## MATERIALS AND METHODS

### Genome analysis and bacterial culturing

The mucin glycan-associated glycosyl hydrolase (GH) families were downloaded from the Carbohydrate-Active enZYmes (CAZy) database (https://www.cazy.org/) and examined as previously described^[25,46-51]^. The mucin-associated GHs include GH33, 16, 29, 95, 20, 2, 35, 42, 98, 101, 129, 89, 85, and 84. Genomes containing one or more copies of a given GH gene were classified as functionally positive for that activity. The proportion of genomes within each species encoding a specific GH function was then calculated using^[25]^:

**Figure:**
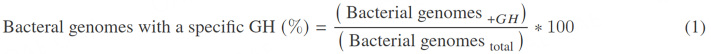


For *in vitro* experiments, we prioritized commercially available strains that could be cultured in a chemically defined growth medium. Accordingly, the following commercially sourced bacterial strains were utilized in this study: *Lactobacillus acidophilus* (*L. acidophilus*) ATCC 4796, *Lactococcus lactis* (*L. lactis*) CB1, *Clostridium symbosium* DSZM (https://www.dsmz.de/) 14940, *Streptococcus thermophilus* (*S. thermophilus*) ATCC 491, *Escherichia coli* (*E. coli*) Nissle DSZM 1917, *Enterococcus faecalis* (*E. faecalis*) Symbioflor DSZM 16431, *Bifidobacterium longum* (*B. longum*) ATCC 55813, *Blautia coccoides* (*B. coccoides*) ATCC 29236, *Blautia producta* (*B. producta*) ATCC 27340D, *Prevotella copri* (*P. copri*) DSZM 18205, *Bacteroides thetaiotaomicron* (*B. thetaiotaomicron*) ATCC 29148, *Bacteroides fragilis* (*B. fragilis*) ATCC 23745, *Bacteroides ovatus* (*B. ovatus*) ATCC 8483, and *A. muciniphila* ATCC BAA-83.

All bacteria were grown anaerobically overnight at 37 °C in an Anaerobe Systems AS-150 anaerobic chamber. *L. acidophilus*, *L. lactis*, and *B. longum* were grown in Man, Rosa, Sharpe (MRS) medium, *C. symbosium*, *S. thermophilus*, *E. coli* Nissle, *E. faecalis*, *B. coccoides*, *B. producta*, *P. copri*, *B. thetaiotaomicron*, *B. fragilis*, and *B. ovatus* were grown in brain heart infusion (BHI) medium supplemented with 1% yeast extract and 0.1% cysteine, and *A. muciniphila* was grown in BHI supplemented with 0.4% porcine gastric mucin. Following verification of bacterial growth, cultures were pelleted by centrifugation at 5,000 ×*g* for 5 min; except for *A. muciniphila* cultures, which were centrifuged at 9,000 ×*g* for 5 min to ensure efficient recovery. In all cases, pellets were washed three times with sterile anaerobic phosphate-buffered saline (PBS) to eliminate residual components of the rich medium. After the final wash, bacteria were resuspended in an equivalent volume of the chemically defined medium ZMB1^[52-54]^ and inoculated into 150 mL of ZMB1 in bioreactors at a starting optical density (OD_600_) of 0.05. Bioreactors were randomly assigned to two groups: (1) vehicle (water) control and (2) MUC2. To the bioreactors receiving mucins, we added 0.5 mg/mL of porcine MUC2 (MyBiosource cat# MBS2028824), and to the control bioreactors, we added the same volume of water. Bioreactors were cultured at 37 °C in biological triplicate under continuous stirring on a magnetic stir plate (200 rpm) to ensure homogeneous mixing. The system operated as a semi-continuous culture, with 10% of the medium replaced daily to maintain nutrient availability. Bioreactors were not pH-controlled, allowing pH to evolve naturally as a function of microbial metabolism. All cultures were maintained entirely within an Anaerobe Systems AS-150 chamber, which provided a stable, oxygen-free environment throughout the experiment. After 72 h of incubation, cultures were centrifuged at 6,000 ×*g* for 5 min to pellet bacteria for genomic DNA (gDNA) extraction, while the resulting cell-free supernatants were sterile-filtered through 0.2 µm syringe filters and subjected to targeted metabolomics analyses. The bacterial pellets were processed for quantitative real time polymerase chain reaction (qPCR).

#### qPCR and calculated colony-forming units

We isolated gDNA from the bacterial pellets using the ZymoBIOMICS DNA isolation kit (Zymo cat# D4300) according to the manufacturer’s instructions with bead beating. qPCR was performed using a Bio-Rad CFX96 Real Time qPCR machine (Bio-Rad) with an initial denaturation at 95°C followed by 40 cycles of 95 °C for 15 s and 60 °C for 30 s. The following forward and reverse primers were used for bacterial species detection:

*Bacteroides* Forward primer: GGTTCTGAGAGGAGGTCCC Reverse primer: CTGCCTCCCGTAGGAGT

*Lactobacillus/Enterococcus* Forward primer: AGCAGTAGGGAATCTTCCA Reverse primer: CACCGCTACACATGGAG

*Bifidobacterium* Forward primer: CTCCTGGAAACGGGTGG Reverse primer: GGTGTTCTTCCCGATATCTACA

*Escherichia* Forward primer: GTTAATACCTTTGCTCATTGA Reverse primer: ACCAGGGTATCTAATCCTGTT

*Prevotella* Forward primer: CCAGCCAAGTAGCGTGCA Reverse primer: TGGACCTTCCGTATTACCGC

*Akkermansia* Forward primer: ACGGGTGGCAGCAGTCGAGA Reverse primer: TGGTTCCGAACAACGCTTGAGACC

Primer specificity was confirmed by melt curve analysis, and amplification efficiencies were validated using standard curves generated from individual cultures of bacteria. All primer sets exhibited efficiencies within the acceptable range (90%-100%) with correlation coefficients (R^2^) > 0.97. Primers and gDNA were added to SYBR Green Master Mix (Genesee Scientific, El Cajon, CA, USA; Cat. No. 17-501DP). Bacterial colony-forming units (CFUs) were calculated from Cycle of Threshold (CT) values based on standard curves of each bacterium^[55]^.

#### Bacterial-conditioned media sample preparations

Prior to analysis, all blank and cell-free bacterial-conditioned ZMBI samples were thawed at ambient temperature. All samples were vortex-mixed for 30 s to ensure homogeneous mixing prior to dilution. Then, in a glass autosampler vial, 2 µL of each cell-free media sample was diluted in a 998 µL of a dilution solution consisting of water: acetonitrile: formic acid (95: 5: 0.1, *v: v: v*), for a 500-fold dilution overall. Subsequently, 2 µL of the diluted sample was injected onto the liquid chromatography-tandem mass spectrometry (LC-MS/MS) system for analysis. The quantitation method filename was specified in the batch file so that the integration of each metabolite peak could be automatically performed after the completion of data acquisition for each blank and media sample.

#### SCIEX QTRAP 6500-based LC-MS/MS system (targeted metabolomics method)

Targeted bioanalysis was performed using an LC-MS/MS system comprised of a Nexera X2 Ultrahigh-Performance Liquid Chromatography (UHPLC) system (Shimadzu, Kyoto, Japan) connected to a QTRAP 6500 system (SCIEX, Framingham, MA, USA). The instrument was operated using Analyst® software (Version 1.6.2; SCIEX), while peak integration and quantitative analysis were performed using the MultiQuant™ software (Version 3.0.1; SCIEX). This system was used to perform the targeted bioanalysis for the Short Chain Fatty Acid (SCFA) Method, the Tyrosine Pathway and Tryptophan Pathway Methods, and the Glutamate Cycle Method for this project (see Supplementary Materials for additional details).

#### SCIEX QTRAP 7500-based LC-MS/MS system (quasi-targeted metabolomics method)

Quasi-targeted bioanalysis was performed using a system consisting of a SCIEX QTRAP 7500 mass spectrometry (MS) system connected to a Shimadzu Series-40 Nexera UHPLC system. The system was operated using SCIEX OS software (Version 3.3.1.43; SCIEX), while peak integration and quantitative analysis was performed using the Analytics Module in SCIEX OS (see Supplementary Materials for additional details).

### Graphs and statistical analysis

All graphs and statistical analyses were performed using GraphPad Prism (version 10.03) software (GraphPad Inc., La Jolla, CA). Comparisons were made with either a two-way analysis of variance (ANOVA) with the Bonferroni post hoc test or Student's *t*-test for data with only two groups. Non-parametric data were log-transformed to pass normality tests before analysis by ANOVA. Differences between the groups were considered significant at P < 0.05.

## RESULTS

**Figure 1:**
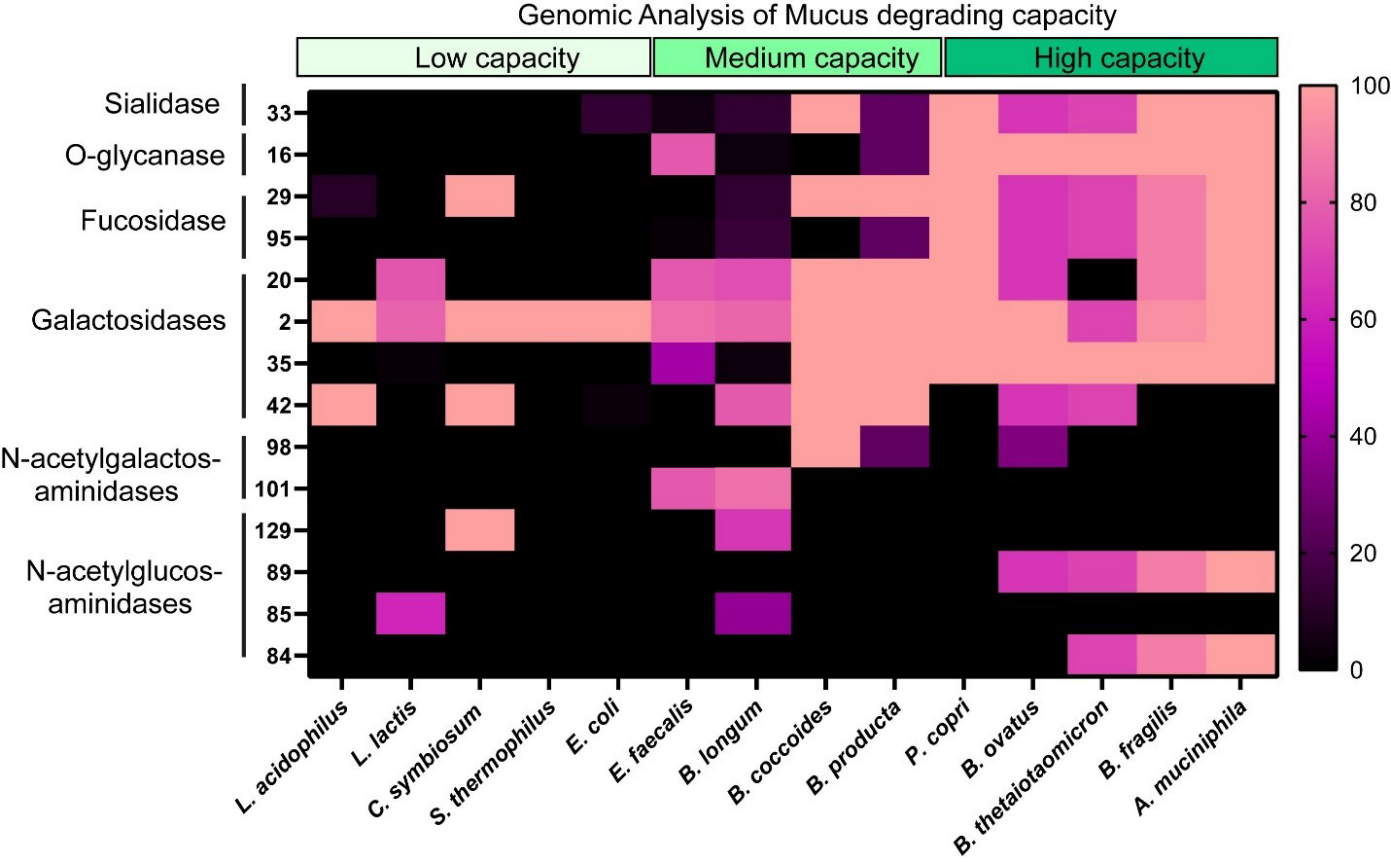
Heat map showing the percentage of bacterial genomes encoding mucin glycan-associated GHs. GH families examined included GH 33, 16, 29, 95, 20, 2, 35, 42, 98, 101, 129, 89, 85, and 84, all of which are involved in mucin glycan degradation. Genome counts and GH annotations were obtained from the CAZy database. Percentages were calculated as the number of genomes containing a given GH family divided by the total number of genomes analyzed for that taxon, multiplied by 100 (See Equation 1). Higher values shown in pink indicate a greater proportion of genomes encoding the specified GH. Genomes analyzed included: *L. acidophilus*, *L. lactis*, *C. symbiosum*, *S. thermophilus*, *E. coli*, *E. feacalis*, *B. longum*, *B. coccoides*, *B. producta*, *P. copri*, *B. ovatus*, *B. thetaiotaomicron*, *B. fragilis*, and *A. muciniphila* genomes. Graph generated using GraphPad Prism software. *L. acidophilus*: *Lactobacillus acidophilus*; *L. lactis*: *Lactococcus lactis*; *C. symbiosum*: *Clostridium symbiosum*; *S. thermophilus*: *Streptococcus thermophilus*; *E. coli*: *Escherichia coli*; *E. feacalis*: *Enterococcus feacalis*; *B. longum*: *Bifidobacterium longum*; *B. coccoides*: *Blautia coccoides*; *B. producta*: *Blautia producta*; *P. copri*: *Prevotella copri*; *B. ovatus*: *Bacteroides ovatus*; *B. thetaiotaomicron*: *Bacteroides thetaiotaomicron*; *B. fragilis*: *Bacteroides fragilis*; *A. muciniphila*: *Akkermansia muciniphila*; GH: glycosyl hydrolase; CAZy: Carbohydrate-Active enZYmes.

**Figure 2:**
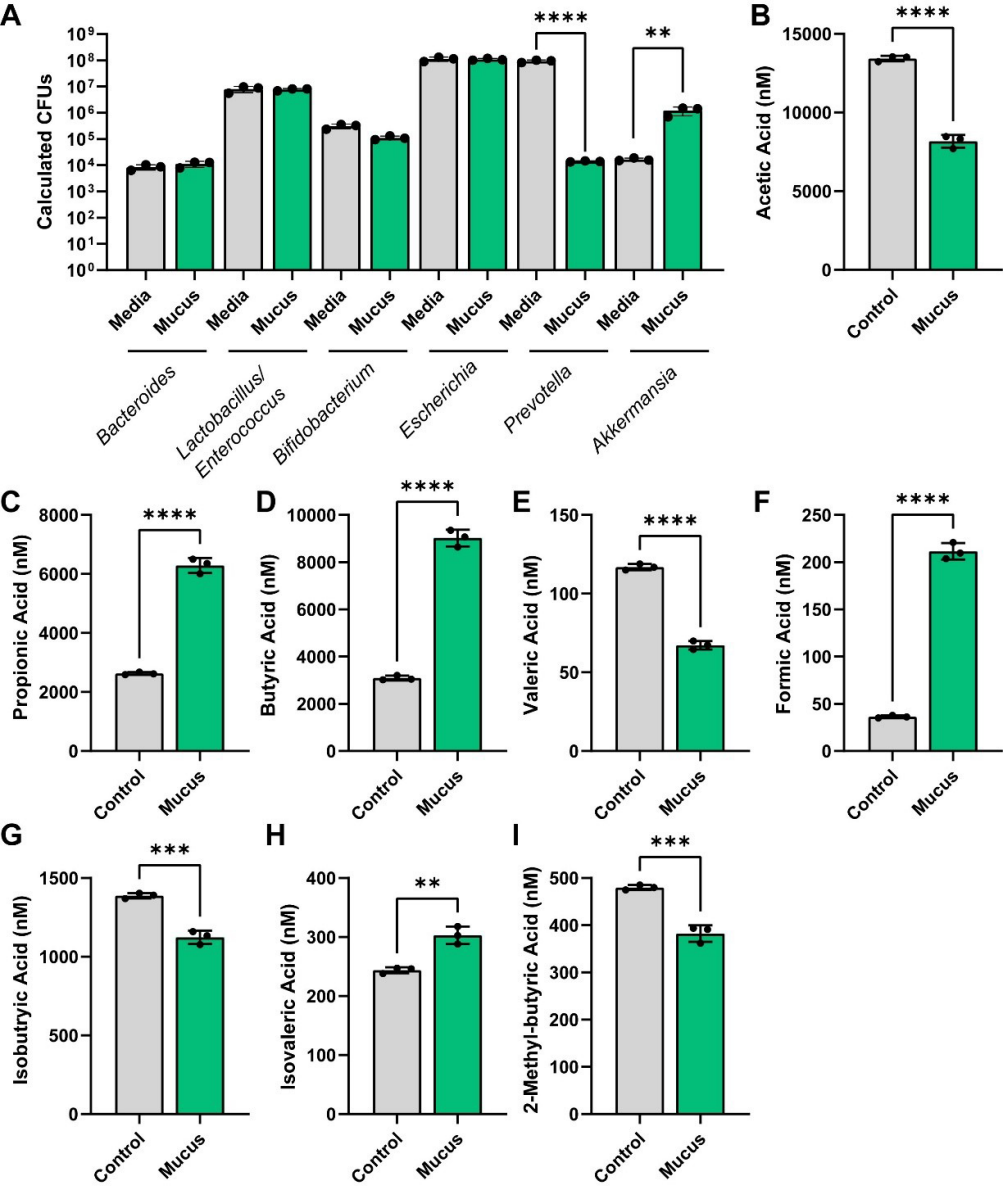
(A) Calculated CFUs of bacterial genera from control and MUC2-supplemented bioreactors after 72 h of growth. Analyzed by two-way ANOVA. (B-I) Individual bar graphs depicting the concentrations (nM) of short-chain fatty acids in cell-free supernatant from control and MUC2-supplemented bioreactors as measured by targeted LC-MS/MS. Bar graphs represent the following compounds: (B) acetic acid (acetate); (C) propionic acid (propionate); (D) butyric acid (butyrate); (E) valeric acid (valerate); (F) formic acid; (G) isobutyric acid (isobutyrate); (H) isovaleric acid (isovalerate); and (I) 2-methyl-butyric acid (2-methyl butyrate). Figure 2A was analyzed using two-way ANOVA, whereas Figure 2B-I was analyzed using the Student's *t*-test. *P* ≤ 0.01 (**), *P* ≤ 0.001 (***), *P* ≤ 0.0001 (****). Each dot represents data from an individual bioreactor (*n* = 3) and each error bar represents the standard deviation. Graphs generated using GraphPad Prism software. CFU: Colony-forming unit; MUC2: Mucin 2; ANOVA: analysis of variance; LC-MS/MS: liquid chromatography-tandem mass spectrometry.

To assess the impact of MUC2 on bacterial metabolism, we assembled a designer community of commensal human gut bacteria with varying mucin-degrading capacities. Mucin-degrading microbes have GHs that target specific glycan structures, including sialic acid (GH33), fucose (GH29, GH95), galactose (GH20, GH2, GH35), N-acetyl-glucosamine (GH84), N-acetyl-galactosamine (GH129 and GH101), and more complex structures (GH16). As representatives of low-capacity mucin glycan degraders, we selected *L. acidophilus*, *L. lactis*, *Clostridium symbiosum* (*C. symbiosum*), *S. thermophilus*, and *E. coli* Nissle, since these strains encode only a limited set of mucin glycan-associated GHs [Figure 1]. For instance, most annotated *L. acidophilus* genomes carried only GH2 and GH4, suggesting that this species is restricted to degrading galactose residues. As our moderate mucin glycan degraders, we selected *E. faecalis*, *B. longum*, *B. coccoides*, *B. producta*, and *P. copri* [Figure 1]. These bacteria harbored > 5 mucin glycan-associated GHs and most strains had the genetic capacity to remove multiple glycan types, including fucose, galactose, and N-acetyl-galactose. For our extensive mucin glycan-degraders, we selected well-characterized members *B. thetaiotaomicron*, *B. fragilis*, *B. ovatus*, and *A. muciniphila* [Figure 1]. These bacteria all possessed > 7 mucin glycan-associated GHs each and had the ability to remove terminal sialic acid and fucose as well as most of the internal sugars [Figure 1]. We grew these bacteria together in anaerobic bioreactors in a chemically defined bacterial medium ZMB1 that promotes the growth of all bacteria. After 3 days of growth, we assessed the bacterial community [Figure 2A]. Although we did not observe significant differences between the OD_600nm_ values of the bioreactors (control: OD_600nm_ 9.1 ± 0.5; MUC2: OD_600nm_ 9.6 ± 0.7), we found that the addition of 0.5 mg/mL porcine MUC2 shifted the bacterial profiles, significantly expanding *Akkermansia* to the detriment of *Prevotella* [Figure 2A]. 

We next sought to examine the functional capacity of these bioreactors in response to mucus. Using targeted LC-MS/MS, we first analyzed the levels of SCFAs. We found that MUC2 significantly reduced acetate levels, but increased the levels of propionate and butyrate [Figure 2B-D]. We also found that MUC2 reduced the production of valerate, iso-butyrate and 2-methyl-butryate, but increased the production of formate and isovalerate [Figure 2E-I]. These data suggest that in a defined human microbial community, mucin degradation supports the production of SCFAs and acids such as propionate, butyrate, isovalerate, and formate.

**Figure 3:**
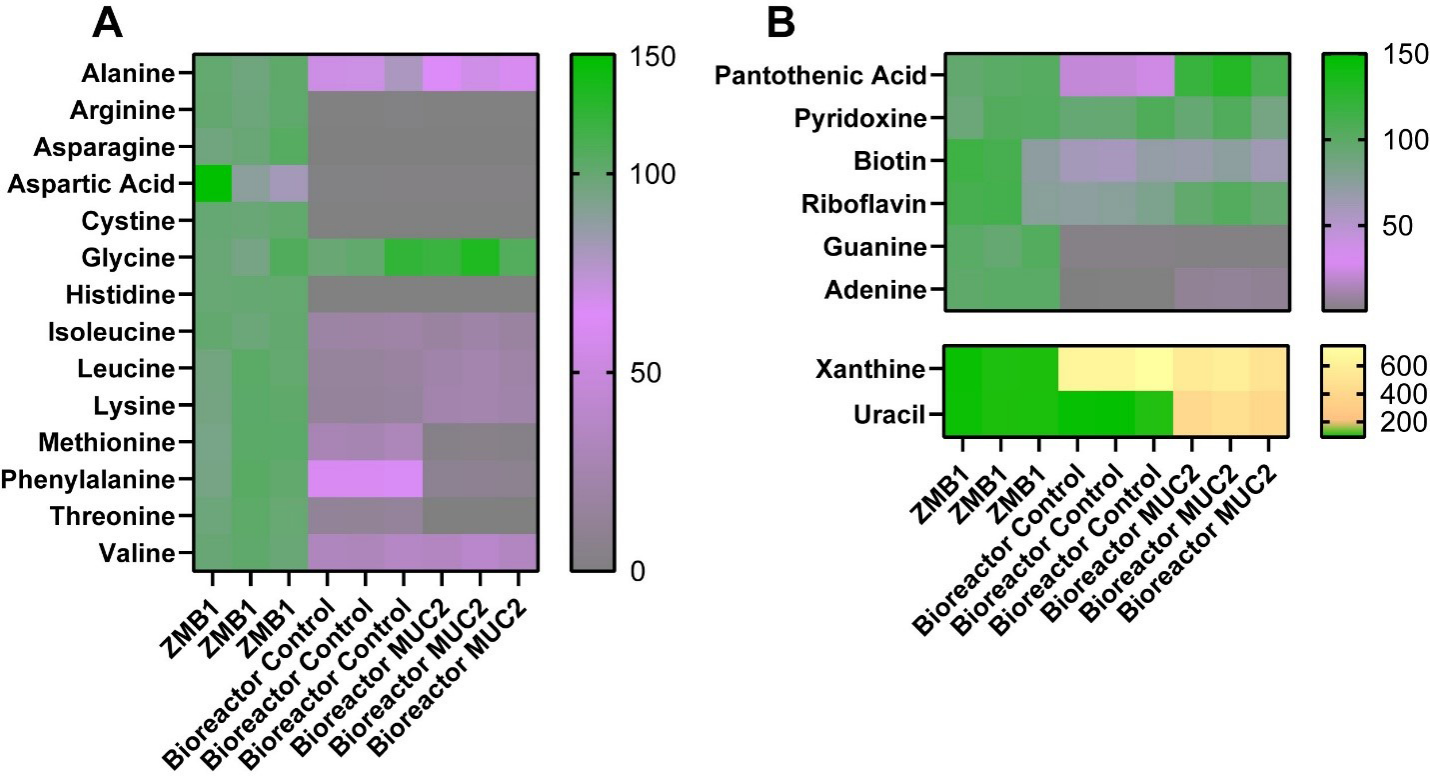
(A) Heat map of the percentage of amino acids in uninoculated ZMB1, control bioreactors, and MUC2-supplemented bioreactors as assessed by LC-MS/MS; (B) Heat map of the percentage of other compounds, including vitamins and nucleic acids, in uninoculated ZMB1, control bioreactors, and MUC2-supplemented bioreactors as assessed by LC-MS/MS. See Table 1 for statistical analysis. Graphs generated using GraphPad Prism software. ZMB1: MUC2: Mucin 2; LC-MS/MS: liquid chromatography-tandem mass spectrometry.

Since our bacterial medium, ZMB1, was chemically defined, we assessed for the microbial consumption of the major components of our media by LC-MS/MS. We found that multiple amino acids were significantly depleted in our bioreactor communities [Figure 3A and Table 1], including alanine, arginine, asparagine, aspartic acid, cystine, histidine, isoleucine, leucine, lysine, methionine, phenylalanine, threonine, and valine. Among these amino acids, we found that MUC2-treated bioreactors had an enhanced reduction in methionine, phenylalanine, and threonine compared to the control bioreactors, suggesting that these microbial communities had an altered consumption of these amino acids. In addition to amino acids, we found that the bioreactor communities, both control and MUC2-treated bioreactors, reduced the levels of riboflavin, biotin, guanine, and adenine compared to the uninoculated medium control [Figure 3B]. Interestingly, the MUC2-treated bioreactors did not reduce the levels of pantothenic acid or riboflavin to the same levels as control bioreactors [Figure 3B]. None of the communities influenced the levels of pyridoxine. However, we found that both control and MUC2-treated bioreactor communities elevated the amount of xanthine, and we found that only MUC2-treated bioreactors elevated the levels of uracil [Figure 3B]. These data suggest MUC2-treated bioreactor communities have an altered metabolism compared to control bioreactor communities. 

**Table 1:** Statistical analysis of the components of ZMB1 in uninoculated medium, control and MUC2-treated bioreactors (two-way ANOVA)

**Compound**	**ZMBI *vs.* Control bioreactors**	**ZMB1 *vs.* MUC2 bioreactor**	**Control *vs.* MUC2 bioreactors**
Alanine	< 0.0001	< 0.0001	0.0966
Arginine	< 0.0001	< 0.0001	0.9975
Asparagine	< 0.0001	< 0.0001	> 0.9999
Aspartic acid	< 0.0001	< 0.0001	0.9998
Cystine	< 0.0001	< 0.0001	0.9965
Glycine	0.4085	0.0054	0.1387
Histidine	< 0.0001	< 0.0001	0.9995
Isoleucine	< 0.0001	< 0.0001	0.9252
Leucine	< 0.0001	< 0.0001	0.2240
Lysine	< 0.0001	< 0.0001	0.0685
Methionine	< 0.0001	< 0.0001	< 0.0001
Phenylalanine	< 0.0001	< 0.0001	< 0.0001
Threonine	< 0.0001	< 0.0001	0.0062
Valine	< 0.0001	< 0.0001	0.7210
Pantothenic acid	< 0.0001	0.0228	< 0.0001
Pyridoxine	0.9867	0.8194	0.8959
Biotin	< 0.0001	0.0002	0.7621
Riboflavin	0.0084	0.9907	0.0118
Guanine	< 0.0001	< 0.0001	0.9976
Adenine	< 0.0001	< 0.0001	0.7494
Xanthine	< 0.0001	< 0.0001	0.0009
Uracil	0.9915	< 0.0001	< 0.0001

ZMB1: MUC2: Mucin 2; ANOVA: analysis of variance.

**Figure 4:**
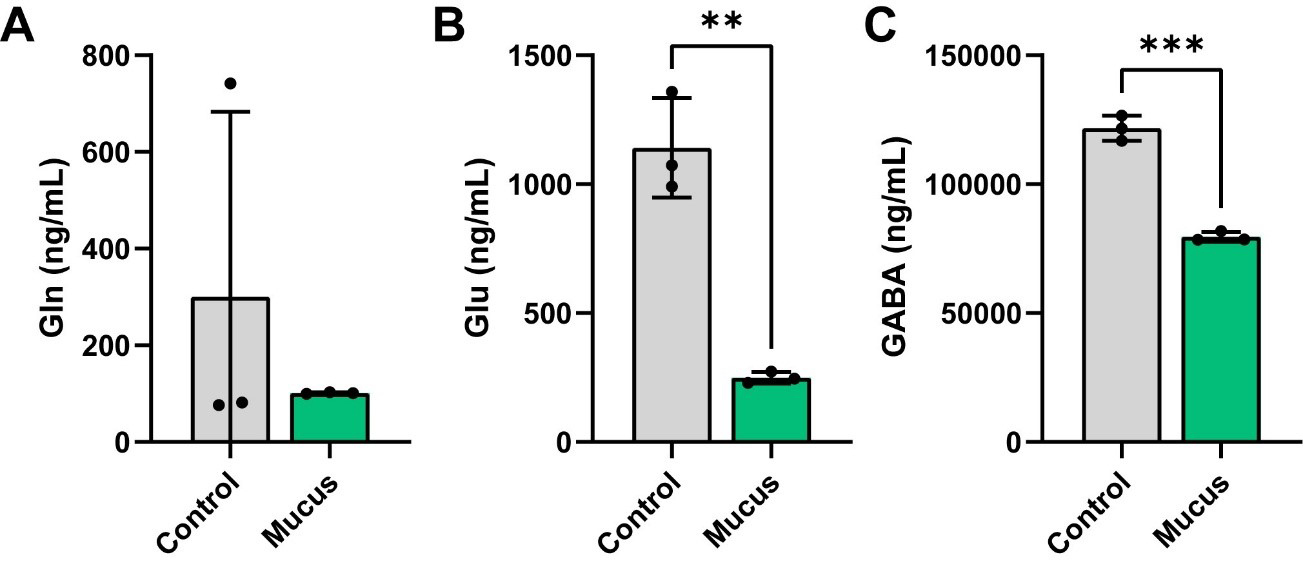
Individual bar graphs depicting the concentrations (ng/mL) of compounds in the Glu/Gln/GABA pathway in cell-free supernatant from control and MUC2-supplemented bioreactors as measured by targeted LC-MS/MS. Bar graphs represent the following compounds: (A) Glutamine; (B) Glutamate; and (C) GABA. Analyzed by student's *t*-test. *P* < 0.05 (*), *P* ≤ 0.01 (**), *P* ≤ 0.001 (***), *P* ≤ 0.0001 (****). Each dot represents data from an individual bioreactor (*n* = 3) and each error bar represents the standard deviation. Student's *t*-test: *P* < 0.05 (*), *P* ≤ 0.01 (**), *P* ≤ 0.001 (***). Graphs generated using GraphPad Prism software. GABA: γ-aminobutyric acid; MUC2: Mucin 2; LC-MS/MS: liquid chromatography-tandem mass spectrometry.

Recent studies have demonstrated that certain gut bacteria can produce neuroactive compounds, such as GABA, levodopa (L-DOPA), and dopamine^[56-64]^. To identify if MUC2 was able to influence neurotransmitter and neuroactive compound production in bacterial communities, we performed targeted LC-MS/MS analysis on our cell-free bacterial supernatants. We first examined the GABA/glutamate (Glu)/glutamine (Gln) cycle in response to MUC2. In this pathway, the amino acid Gln can be converted into Glu, and Glu can then be subsequently converted into the neurotransmitter GABA. We found that the levels of Gln were unchanged by presence of MUC2 in the bacterial communities [Figure 4A]. However, we observed a significant reduction in the levels of Glu and GABA in the MUC2-treated bioreactors [Figure 4B and C], suggesting that MUC2 influences the consumption of Glu and the production of GABA. 

**Figure 5:**
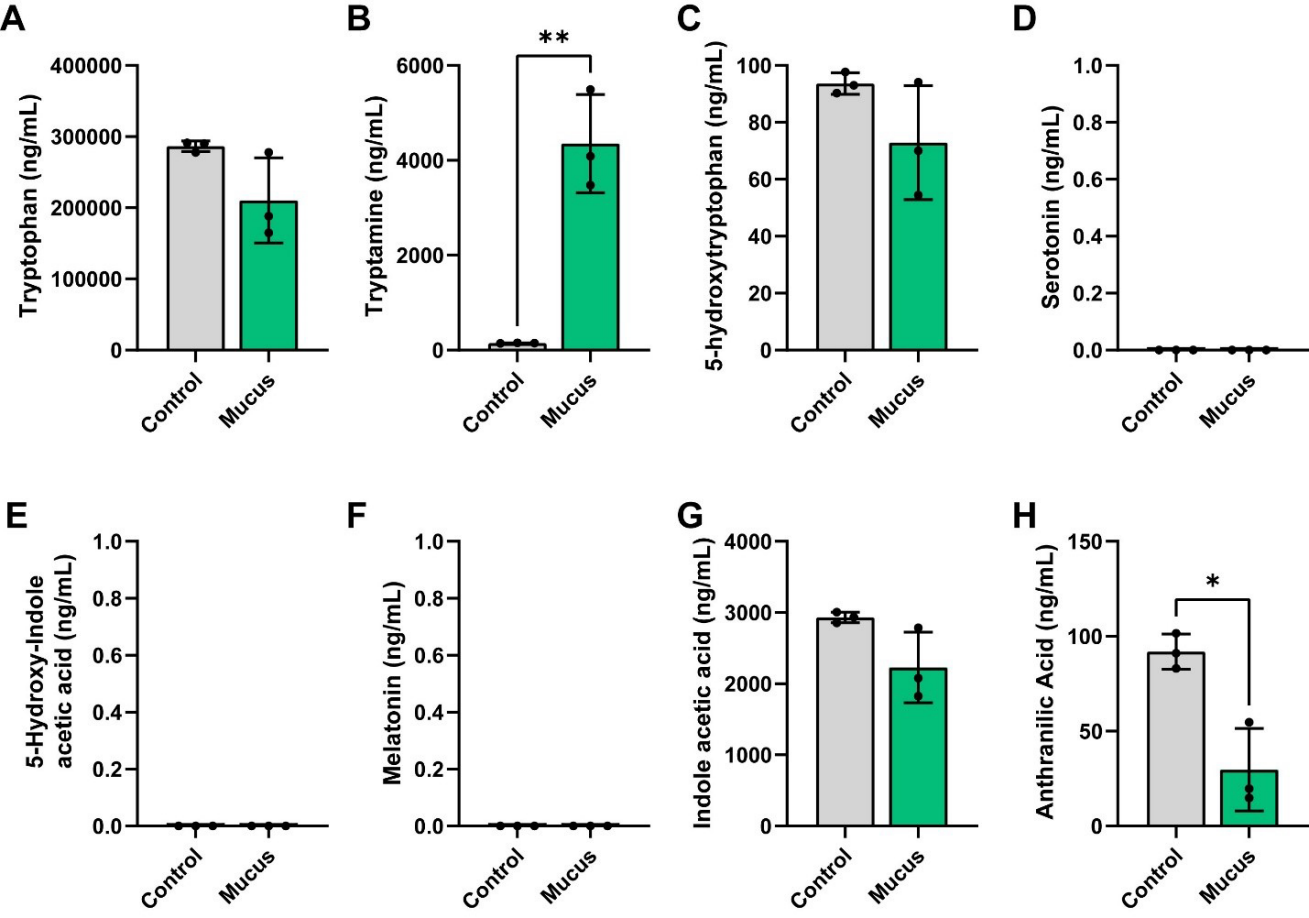
Individual bar graphs depicting the concentrations (ng/mL) of compounds in the tryptophan pathway in cell-free supernatant from control and MUC2-supplemented bioreactors as measured by targeted LC-MS/MS. Bar graphs represent the following compounds: (A) Tryptophan; (B) Tryptamine; (C) 5-hydroxytryptophan (5-HTP); (D) 5-hydroxytryptamine (5-HT, serotonin); (E) 5-hydroxy-indole acetic acid; (F) melatonin; (G) indole acetic acid and (H) anthranilic acid. Analyzed by Student's *t*-test. *P* < 0.05 (*), *P* ≤ 0.01 (**), *P* ≤ 0.001 (***), *P* ≤ 0.0001 (****). Each dot represents data from an individual bioreactor (*n* = 3) and each error bar represents the standard deviation. Student's *T*-test: *P* < 0.05 (*), *P* ≤ 0.01 (**). Graphs generated using GraphPad Prism software. MUC2: Mucin 2; LC-MS/MS: liquid chromatography-tandem mass spectrometry.

We next examined the tryptophan pathway. In this pathway, the amino acid tryptophan can be metabolized into tryptamine. We found that although tryptophan levels were not significantly different between the groups, we did observe elevated production of tryptamine in our bioreactors supplemented with MUC2 [Figure 5A and B]. Tryptophan can also be converted to 5-hydroxytryptophan (5-HTP), which is subsequently converted into serotonin [5-hydroxytryptamine (5-HT)]. Serotonin can then be degraded into 5-hydroxyindoleacetic acid (5-HIAA) or converted to melatonin. Interestingly, we did not observe any difference between the levels of 5-HTP between the bioreactors [Figure 5C] and we did not detect any serotonin, 5-HIAA, or melatonin [Figure 5D-F]. Tryptophan can be degraded by some bacteria into indoles, such as indoleacetic acid or anthranilic acid. Although we did not find any differences in indoleacetic acid production, we did observe lower concentrations of anthranilic acid in our communities supplemented with MUC2 [Figure 5G and H]. These data suggest that MUC2 elevates tryptamine and reduces anthranilic acid levels in defined microbial communities. 

**Figure 6:**
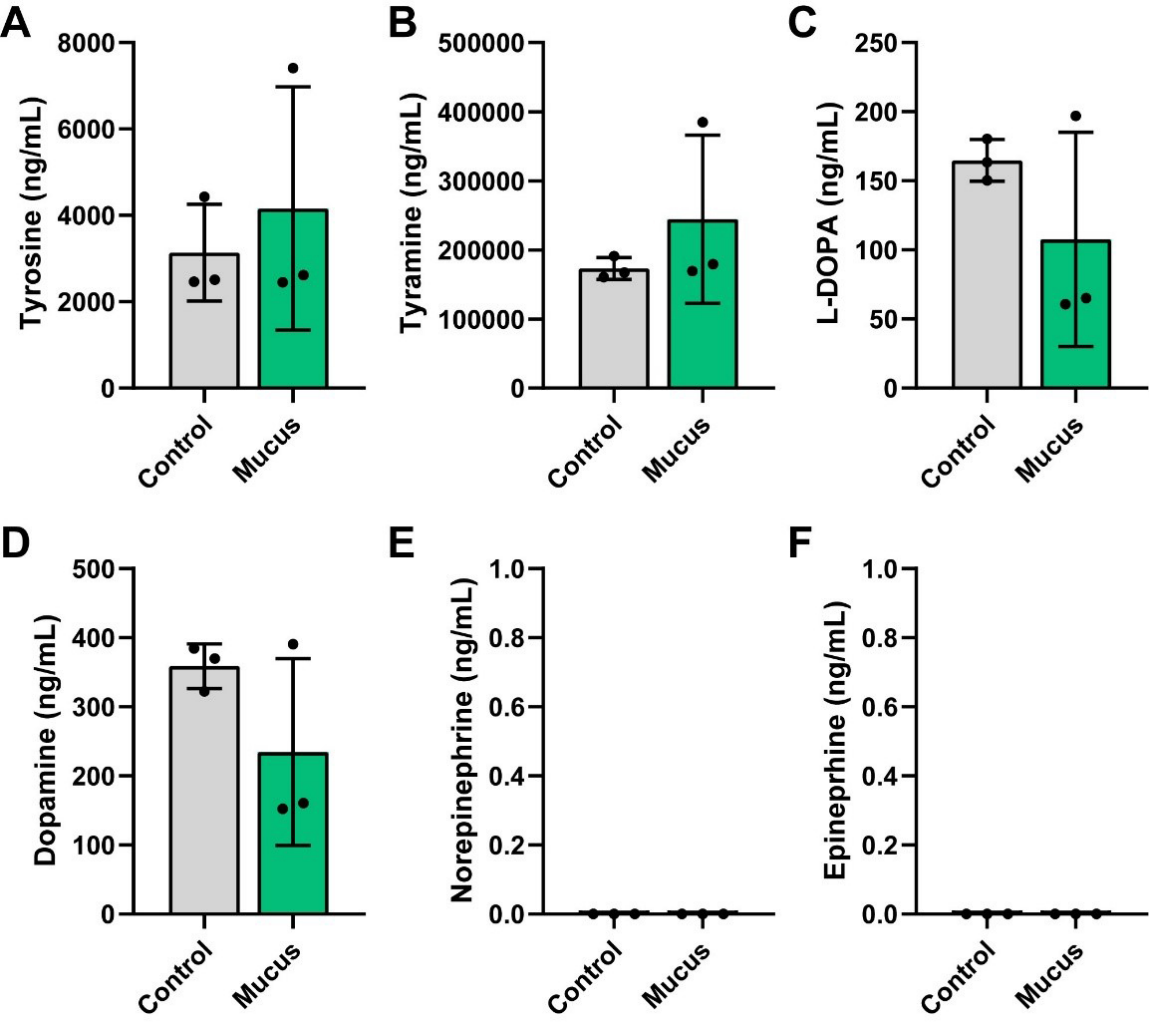
Individual bar graphs depicting the concentrations (ng/mL) of compounds in the tyrosine pathway in cell-free supernatant from control and MUC2-supplemented bioreactors as measured by targeted LC-MS/MS. Bar graphs represent the following compounds: (A) Tyrosine, (B) Tyramine, (C) L-DOPA, (D) Dopamine, (E) Norepinephrine, and (F) Epinephrine. Analyzed by Student's *t*-test. *P* < 0.05 (*), *P* ≤ 0.01 (**), *P* ≤ 0.001 (***), *P* ≤ 0.0001 (****). Each dot represents data from an individual bioreactor (*n* = 3) and each error bar represents the standard deviation. Student's *t*-test. Graphs generated using GraphPad Prism software. MUC2: Mucin 2; LC-MS/MS: liquid chromatography-tandem mass spectrometry; L-DOPA: levodopa.

We also examined compounds in the tyrosine pathway. In this pathway, the amino acid tyrosine can be converted to tyramine or converted to L-DOPA and subsequently to dopamine, norepinephrine and ultimately to epinephrine. Interestingly, we did not observe any differences in the levels of tyrosine, tyramine, L-DOPA, or dopamine [Figure 6A-D] and we did not detect any norepinephrine or epinephrine [Figure 6E and F]. Collectively, these data suggest that MUC2 significantly impacts bacterial metabolism and metabolite production, including the consumption of amino acids and generation of SCFAs and neuroactive compounds such as GABA, Glu, tryptamine, and anthranilic acid. 

## DISCUSSION

The intestinal mucus layer represents a dynamic interface between host and microbes, providing both a barrier against micro-organisms and a nutrient reservoir for specialized commensals. In this study, we systematically evaluated the impact of MUC2 supplementation on a defined microbial consortium with varying mucin-degrading capacities. By reconstructing a simplified, yet functionally diverse community, we demonstrated that mucus is a key driver of microbial composition and function. The expansion of *A. muciniphila* in the presence of MUC2 highlights the competitive advantage afforded to specialized mucin degraders. These findings align with *in vivo* observations where *A. muciniphila* abundance increases during mucus-rich states^[36]^, and they support the notion that mucus composition strongly influences microbiota structure.

Beyond shaping microbial membership, MUC2 supplementation profoundly altered the metabolic output of the community. We observed enhanced production of propionate, butyrate, isovalerate, and formate, coupled with a reduction in acetate. These changes are consistent with cross-feeding dynamics in the literature, where acetate production by mucin degraders can cross-feed non-mucin degraders and stimulate propionate and butyrate production. For example, addition of mucin-degrading microbes - such as *A. muciniphila*, *B. thetaiotaomicron*, *B. vulgatus*, and *Ruminococcus obeum* - to antibiotic-treated stool seeded human intestinal microbial ecosystems (M-SHIME) containing mucus was found to increase the production of propionate^[44]^. Additionally, mucin degradation by *A. muciniphila* has been shown to cross-feed gut commensal microbes *Anaerostipes caccae* (*A. caccae*)*, Eubacterium hallii*, and *Faecalibacterium prausnitzii* and promote the production of butyrate^[39-42]^. *A. muciniphila* has also been shown to release sialic acid from mucin and promote butyrate production by co-cultured Clostridia, such as *Roseburia inulinivorans*, *Roseburia intestinalis*, *Ruminococcus faecis*, *Agathobacter rectalis*, and *Faecalibacterium prausnitzii*^[43]^. These studies and our own data suggest that mucin-degrading bacteria can release glycans and generate metabolites such as acetate that can promote cross-feeding of other microbes and the generation of butyrate. Butyrate is a critical metabolite for host health, serving as an energy source for colonocytes and regulating epithelial barrier integrity^[65]^. Although we do not know which bacteria in our community are responsible for our observed increase in butyrate production, from genome analysis we predict that *C. symbiosum* and *Blautia* species are likely contributors to butyrate production. Additional experiments, including selective removal of these taxa from the defined community, will be required to confirm the butyrate-producing organisms.

Interestingly, some studies suggest that non-degrading microbes can enhance the mucin-degrading capacity of specialists. For example, co-culture of non-mucin degrading *A. caccae* with *A. muciniphila* increased the expression of mucin-glycan degradation genes in *A. muciniphila*. This indicates that interactions with neighboring microbes can stimulate the production of mucin-degrading enzymes^[42]^. We did not assess the mucin-degrading enzymes in this study; however, we think this is an interesting component to include in future studies. Our findings extend prior pairwise co-culture studies by showing that mucin-driven cross-feeding can modulate the collective SCFA profile of a complex community, with implications for host physiology.

In addition to shaping SCFAs, we found that MUC2 shaped amino acid consumption and the production of neuroactive compounds. The MUC2-treated bioreactor communities had an enhanced utilization of the amino acids methionine, phenylalanine, and threonine. In microbial communities, methionine, phenylalanine, and threonine can serve as substrates for several metabolic pathways. Methionine can be used to generate hydrogen sulfide^[66]^ and hydrogen sulfide has been shown to stimulate colonic mucus production^[67]^, providing a potential feedback loop to promote mucus homeostasis after mucus degradation. Phenylalanine can be degraded by gut bacteria into aromatic metabolites such as phenylacetic acid^[68]^ and p-cresol^[69]^, compounds that can cross-feed other microbes or impact host physiology. Threonine can also be metabolized to generate propionate^[70]^. Consistent with the lower levels of threonine in the MUC2-treated bioreactors, we did observe higher levels of propionate. It is possible that the bacteria used more threonine in the medium to generate more propionate. Threonine can also be converted to glycine and acetyl-CoA^[71]^, supporting both energy metabolism and biosynthetic pathways. We found the same levels of glycine in our bioreactors as in the original ZMB1 medium. It is possible that the bacterial communities did not use the available glycine, or that the bacteria simply replaced the glycine by converting threonine into glycine. We did not measure all the potential metabolites that were in the bioreactors, but we suspect that future studies using non-targeted LC-MS/MS would be valuable for identifying other mucin-associated metabolites.

One interesting finding in this study was that MUC2 treatment reduced the concentrations of Glu, GABA, and anthranilic acid. GABA is a particularly important neurotransmitter in the gut as it regulates intestinal motility, secretion, visceral nociception, and cytokine production^[58,72-77]^. Several bacteria can generate GABA, including *Lactobacillus, Lactococcus, Bacteroides*, and *Bifidobacterium* species^[58-60,64,78-86]^. GABA production is commonly regulated by environmental pH and acts as a protective mechanism for these bacteria^[59,82,87]^. We did not measure the pH of the bioreactors, but it is possible that the MUC2-treated communities may have a pH that is not conducive to GABA production^[82]^. Additionally, carbohydrates are known to impact GABA production^[83,88-90]^. For example, many bacteria preferentially use glucose over alternative carbohydrate sources^[91]^. It is also possible that the increased availability of mucin-derived glycans in the MUC2 bioreactors could have shifted carbon utilization away from GABA production and towards other pathways. GABA production in *Limosilactobacillus fermentum* is regulated by the quorum-sensing molecule AI-2^[92]^, and mucin glycans are known to influence quorum-sensing pathways in several bacteria^[93-96]^. As a result, another possibility is that MUC2 glycans influence quorum sensing, which in turn regulates GABA production. Another option is that MUC2 glycans or bacterial cross-feeding may reduce the *de novo* synthesis of Glu, which is the precursor and rate-limiting compound for GABA production. In addition to being synthesized, GABA can be used by some bacteria as a carbon and nitrogen source^[97,98]^. Certain bacteria, such as *E. coli*, can convert GABA to Glu and succinate^[99,100]^. Another interpretation is that bacteria such as *E. coli* Nissle in our bioreactors may have consumed more GABA in the MUC2-treated bioreactors than in the control bioreactors. A time course analysis of GABA and the inclusion of pH measurements, bacterial RNA sequencing and examination of quorum-sensing compounds would be helpful in future work to identify the mechanisms that regulate GABA. 

In addition to regulating GABA, we found that MUC2 elevated the levels of tryptamine. Certain gut bacteria, including *Ruminococcus gnavus*, *Clostridium sporogenes*, *Enterocloster asparagiformis*, *Blautia hansenii*, and *Clostridium nexile*, can convert dietary tryptophan to tryptamine^[63,101-103]^. Tryptamine has a similar structure to serotonin (5-HT); as a result, tryptamine can act as an agonist for intestinal serotonin receptors and trace amine-associated receptors^[104-107]^. In this study, we did not examine how the bacterial metabolites affected the gut epithelium. However, based on their profiles, we postulate that mucus may alter microbial contributions to host neuroactive signaling.

In this study, we used porcine MUC2, but it should be noted that mucin glycosylation is host-specific. Although both porcine and human mucins share conserved features, such as heavily O-glycosylated glycans on core structures and similar glycosyltransferase pathways, there are differences in the dominant core structures and terminal glycans. For example, mass spectrometry-based studies of pig and human gastric mucins show that porcine mucins are dominated by extended core-1 and core-2 structures with variable glycan length and sulphation, whereas extended core-3 and core-4 structures are rare^[108-110]^. Pig gastric mucins also contain galactose-terminated glycans with few terminal N-acetyl-neuraminic acid residues. In contrast, human mucins are enriched for extended core-2 glycans and exhibit greater terminal fucosylation and minimal sulphation^[108,111]^. In the colon, porcine colonic mucus contains cores 1, 2, 3, and 4, with a predominance of core 4 structures and has an equal distribution of N-acetylneuraminic acid and N-glycolylneuraminic acid^[112,113]^. In contrast, human colonic mucus contains cores 1-5 and is dominated by core 3 structures^[14,114,115]^. Additionally, humans are unable to make N-glycolylneuraminic acid and the glycans are commonly terminated by N-acetylneuraminic and sulfate residues^[14,114,115]^. These studies highlight fundamental differences in terminal epitope composition that could impact the way microbes interact with the mucin. In the future, it would be advantageous to determine how defined microbial communities interact with human MUC2. We speculate that supplementation with human MUC2, which is enriched for core-3 glycans and terminal fucosylation with minimal sulphation, might shift cross-feeding networks and metabolic outputs, potentially enhancing utilization by fucose-adapted commensals.

To dissect mucus-microbial interactions, we intentionally employed a simplified, defined microbial consortium rather than a highly complex community. Consortium members were selected to represent a spectrum of mucin-degrading capacities, including robust primary degraders (*A. muciniphila*, *B. thetaiotaomicron*, *B. ovatus*, *B. fragilis*), taxa with partial or context-dependent mucin utilization (*B. producta*, *B. coccoides*, *B. longum*, *E. faecalis*, *P. copri*), and organisms lacking canonical mucin-degrading enzymes that may function as secondary consumers (*L. acidophilus*, *L. lactis*, *S. thermophilus*, *C. symbiosum*, and *E. coli*). This design allowed us to isolate the effects of mucin availability on community structure and metabolite output while minimizing confounding variables inherent to more complex systems. We focused on microbes that were commercially available, well characterized, and supported by high-quality reference genomes. In the future, we would like to include the other abundant gut taxa such as *Eubacterium*, *Roseburia* and *Faecalibacterium*, which are important contributors to short-chain fatty acid production^[116-121]^, as well as other mucin glycan degraders such as *Ruminococcus gnavus*, *Ruminococcus torques*, *Parabacteroides distasonis*, and non-toxigenic *Clostridium perfringens*^[25,32,38,122,123]^. We believe our findings establish a framework for understanding how mucins shape microbial metabolite production, and we hope it motivates future studies incorporating larger and more complex communities to better approximate the native gut ecosystem.

While this study provides new insight into how mucin degradation shapes microbial communities and metabolism, several limitations should be acknowledged. First, our defined consortium, though designed to capture a range of mucin-degrading capacities, cannot fully recapitulate the taxonomic and functional complexity of the human gut microbiota. In particular, the exclusion of keystone fermenters, such as *Eubacterium*, *Roseburia*, and *Faecalibacterium*, limits the extent to which the data can be directly extrapolated to the *in vivo* gut ecosystem. Second, the use of porcine-derived MUC2 may not perfectly mirror the glycosylation patterns of human mucins, which could influence bacterial utilization and cross-feeding dynamics. Moreover, purified MUC2 represents a simplified substrate relative to the complex mixture of proteins and lipids present in the mucus layer of the human colon^[123]^. Third, the bioreactor model provides a controlled environment that facilitates mechanistic interrogation; however, it lacks host factors such as epithelial responses, immune signaling, and mucus turnover that influence microbial ecology *in vivo*. Finally, although metabolomic profiling revealed significant shifts in amino acid, SCFA, and neuroactive compound levels, these data cannot be directly attributed to specific taxa without isotopic tracing or genetic knockouts. Additionally, since we observed only minimal changes in our community composition, we could not definitively correlate our findings with any specific bacterial groups. Addressing these limitations in future *in vivo* studies will be critical to determine the physiological consequences of mucus-driven microbial metabolism.

Our findings highlight mucus as a central ecological driver in the gut ecosystem. By supplementing defined microbial communities with MUC2, we demonstrated that mucus profoundly alters both bacterial composition and metabolic output, promoting the expansion of mucin-degrading specialists and influencing the production of SCFAs, amino acid derivatives, and neuroactive metabolites. These results underscore the dual role of mucin degradation not only as a nutrient acquisition strategy for specialized microbes, but also as a key mechanism of cross-feeding that sustains broader community function. Given that microbial metabolites directly influence epithelial integrity, immune responses, and gut-brain communication, our data suggest that shifts in mucus utilization may have far-reaching implications for host physiology. While our defined bioreactor system does not capture the full complexity of the human gut, it provides a powerful platform for mechanistic dissection of mucus-microbiota interactions under controlled conditions. Ultimately, this work establishes a foundation for future studies that link mucus-driven metabolic networks to host health and disease, advancing our understanding of how the mucus barrier shapes the intestinal ecosystem.

## DECLARATIONS

### Availability of data and materials

The datasets generated from the study are available from the corresponding authors upon reasonable request.

### Copyright

© The Author(s) 2026.
