# Supplementary material for: MUC2 shifts the metabolic profiles of commensal bacteria in defined microbial communities
Source: Microbiome Res Rep. 2026 Jan 21;5(1):1. doi: 10.20517/mrr.2025.91 (PMC13091087; doi:10.20517/mrr.2025.91)
Supplement: Supplementary file 1 [file mrr-5-1-1-SupplementaryMaterials.pdf]

Supplementary Materials

MUC2 shifts the metabolic profiles of commensal bacteria in defined microbial communities

Erin Chard[1], Adelaide E. Horvath[1,2,3], Makenna Grozis[1,4], Renata Rocha do Nascimento[1], Paul R.S. Baker[5], Santosh Kapil Kumar Gorti[5], Robert Proos[5], Thomas D. Horvath[6,7,8], Melinda A. Engevik[1,9]

[1]Department of Regenerative Medicine & Cell Biology, Medical University of South Carolina, Charleston, SC 29425, USA.

[2]Department of Biology & Biochemistry, University of Houston, Houston, TX 77204, USA.

[3]Department of Mathematics, University of Houston, Houston, TX 77204, USA.

[4]Department of Biology, Elon University, Elon, NC 27244, USA.

[5]Metabolomics & Lipidomics Applications, SCIEX, Marlborough, MA 01752, USA.

[6]Department of Pathology & Immunology, Baylor College of Medicine, Houston, TX 77030, USA.

[7]Department of Pathology, Texas Children's Hospital, Houston, TX 77030, USA.

[8]Department of Pharmacy Practice & Translational Research, College of Pharmacy, University of Houston, Houston, TX 77030, USA.

[9]Department of Pharmacology & Immunology, Medical University of South Carolina, Charleston, SC 29425, USA.

Correspondence to: Dr. Thomas Horvath, Department of Pathology, Texas Children's Hospital, Houston, TX 77030, USA. E-mail: Thomas.Horvath2@bcm.edu; Dr. Melinda A. Engevik, Department of Regenerative Medicine & Cell Biology, Medical University of South Carolina, Charleston, SC 29425, USA. E-mail: engevik@musc.edu

Chemicals, Reagents, and Consumables

Optima™ liquid chromatography-mass spectrometry (LC/MS)-grade water, methanol, and acetonitrile were purchased from Fisher Scientific (Waltham, MA, USA). Mobile phase

modifiers, including mass spectrometry (MS)-grade ammonium formate and heptafluorobutyric acid, were purchased from Millipore-Sigma (Burlington, MA, USA). Metabolite reference standards contained in the one of the new chemically defined media in reference 52 (ZMB1) growth media were purchased for the amino acids (alanine, arginine, asparagine, aspartic acid, cysteine, cysteine, glutamine, glutamate, glycine, histidine, isoleucine, leucine, lysine, methionine, phenylalanine, proline, threonine, tryptophan, tyrosine, and valine), the vitamins (biotin, folic acid, nicotinamide, pantothenic acid, pyridoxine, riboflavin and thiamine), the nucleic acids (adenine, guanine, uracil), sugars (glucose and inositol), and lipoic acid and glutathione were purchased from Millipore-Sigma.

For the derivatization procedures used in the Short Chain Fatty Acid (SCFA) Method, the 1-(3-dimethylaminopropyl)-3-ethylcarbodiimide hydrochloride (EDAC), 2-mercaptoethanol, and succinic acid were each purchased from Fisher Scientific, and the aniline and [$^{13}C_6$]-aniline [for carbon-13 labeled internal standard (IS) synthesis] were each purchased from Millipore-Sigma. For the preparation of the unlabeled analytical standards and the [$^{13}C_6$]-labeled IS compounds, unlabeled Optima-grade formic acid and acetic acid were purchased from Fisher Scientific, and unlabeled propionic acid, isobutyric acid, butyric acid, 2-methylbutyric acid, isovaleric acid, valeric acid, and hexanoic acid reference standards were all purchased from Millipore-Sigma. A 5-µm Viva biphenyl (BiPh; 100 mm × 1 mm, 300 Å pore) analytical column and a 5-µm Viva BiPh (10 mm × 2.1 mm) guard column were purchased from Restek (Bellefonte, PA, USA).

For the Glutamate Cycle Method, unlabeled γ-aminobutyric acid (GABA), L-glutamate, and L-glutamine analytical standards, and d6-GABA, d5-L-glutamate, and d5-L-glutamine deuterated IS compounds, and a 2.7-µm Supelco Ascentis Express HILIC (150 mm × 2.1 mm, 90 Å pore) analytical column were all purchased from Millipore-Sigma. For the Tryptophan Pathway Method, unlabeled N-acetylserotonin and 5-hydroxyindole-3-acetic acid (5-HIAA) were purchased from Millipore-Sigma, and unlabeled 5-hydroxytryptophan, melatonin, serotonin hydrochloride, and L-tryptophan analytical standards were all purchased from Fisher Scientific. Deuterated Internal Standards (IS) compounds, including d5-5-HIAA, d3-5-hydroxytryptophan, d_4-melatonin and d5-L-tryptophan, were all purchased from CDN Isotopes (Pointe Claire, Quebec, Canada), and d_4-serotonin hydrochloride was purchased from Santa Cruz Biotechnology

(Dallas, TX, USA). A 3-µm Luna C18(2) (150 mm × 1 mm, 100 Å pore) analytical column and a Security Guard C18 (4 mm × 2 mm) guard column were purchased from Phenomenex (Torrance, CA, USA).

For the Tyrosine Pathway Method, unlabeled L-tyramine was purchased from Fisher Scientific, and unlabeled anthranilic acid, dopamine hydrochloride, epinephrine, levodopa (L-DOPA), D,L-norepinephrine, and L-tyrosine analytical standards were purchased from Millipore-Sigma. Deuterated IS compounds, such as d_4-dopamine, d6-epinephrine and d3-L-DOPA, were all purchased from Millipore-Sigma, and d_4-L-tyramine was purchased from Santa Cruz Biotechnology (Dallas, TX, USA). A 2.7-µm Raptor C18 (100 mm × 1 mm, 90 Å pore) analytical column and a Restek Ultra C18 (10 mm × 2.1 mm, 100 Å pore) guard column were purchased from Restek.

Targeted SCFA Method

The derivatization procedures and LC-MS/MS method conditions for the targeted SCFA Method have been described previously[37-40]. The final sample preparation procedure included the dilution of 10 µL of derivatized bioreactor media sample in 90 µL of an Internal Standard Solution - A (ISS-A) (Dilution factor, DF, = 37.4-fold overall for each sample) that contained a concentration of 2,500 nM each for the carbon-13-labeled IS compound derivatives including [$^{13}C_6$]-N-phenyl formamide, [$^{13}C_6$]-N-phenyl acetamide, [$^{13}C_6$]-N-phenyl propanamide, [$^{13}C_6$]-N-phenyl isobutanamide, [$^{13}C_6$]-N-phenyl butanamide, [$^{13}C_6$]-N-phenyl 2-methylbutanamide, [$^{13}C_6$]-N-phenyl isopentanamide, [$^{13}C_6$]-N-phenyl pentanamide, and [$^{13}C_6$]-N-phenyl hexanamide in a solution of acetonitrile:water (2:8, vol/vol). 4 µL of each sample was injected onto the QTRAP 6500 system (SCIEX, Framingham, MA, USA) for bioanalysis. The linear dynamic range for the method was 9.77 nM – 10,000 nM for each of the SCFA derivatives including N-phenyl formamide, N-phenyl acetamide, N-phenyl propanamide, N-phenyl isobutanamide, N-phenyl butanamide, N-phenyl 2-methylbutanamide, N-phenyl isopentanamide, N-phenyl pentanamide, and N-phenyl hexanamide.

Targeted Tyrosine Pathway Method

The critical solution preparations and LC-MS/MS conditions for the targeted Tyrosine Pathway Method have been described previously[37-40]. Briefly, 10 µL of conditioned growth media was diluted in 90 µL of an ISS-A solution (DF = 10-fold for each sample) that contained IS concentrations of 250 ng/mL for d_4-tyramine, 1,000 ng/mL for d_3-L-DOPA, 125 ng/mL for d_4-dopamine, and 200 ng/mL for d_6-epinephrine and each sample was vortex-mixed for ~30 seconds and transferred to an autosampler vial. 10 µL of each sample was injected onto the QTRAP 6500 system for bioanalysis. The linear dynamic range for the method was 0.977 ng/mL – 1,000 ng/mL for the following unlabeled metabolites: tyramine, dopamine, L-DOPA, tyrosine, norepinephrine, epinephrine, anthranilic acid, and quinolinic acid.

Targeted Tryptophan Pathway Method

The critical solution preparations and LC-MS/MS conditions for the targeted Tryptophan Pathway Method have been described previously[37-40]. Briefly, 10 µL of conditioned growth media was diluted in 90 µL an ISS-A solution (DF = 10-fold for each sample) that contained IS concentrations of 500 ng/mL for d5-tryptophan, d_4-serotonin and d_4-melatonin, and 1,500 ng/mL for d5-5-HIAA and each sample was vortex-mixed for ~30 seconds and transferred to an autosampler vial. 10 µL of each sample was injected onto a QTRAP 6500- LC-MS/MS system for bioanalysis. The linear dynamic range for the method was 0.977 ng/mL – 1,000 ng/mL for the following unlabeled metabolites: tryptophan, serotonin, melatonin, 5-HIAA, 5-hydroxytryptophan, N-acetylserotonin, tryptamine, and indoleacetic acid.

Targeted Glutamate Cycle Method

The critical solution preparations and LC-MS/MS conditions for the targeted Glutamate Cycle Method have been described previously[37-40]. Briefly, 10 µL of conditioned growth media was diluted in 90 µL of an ISS-A solution (DF = 10-fold for each sample) that contained IS concentrations of 500 ng/mL for d6-GABA, d5-glutamate and d5-glutamine and each sample was vortex-mixed for ~30 seconds and transferred to an autosampler vial. 10 µL of each sample was injected onto the QTRAP 6500 system for bioanalysis. The linear dynamic range for the method was 0.977 ng/mL – 1,000 ng/mL for the following unlabeled metabolites: GABA, glutamate and glutamine.

Critical Solution Preparations for the Individual ZMBI Components for the Quasi-Targeted Metabolomics (QT-Meta) Method

The LC-MS/MS-based QT-Meta Method described here is based on a commercially developed SCIEX Method (Targeted Profiling Method for Metabolomics Technical Report)[41] that was bundled with the QTRAP 7500 purchase. To ensure complete coverage of the metabolites used in ZMBI growth medium preparations, the molecule-specific multiple-reaction monitoring (MRM) parameters were optimized for each ZMBI component in positive and negative ion modes on the QTRAP 7500 system; all other metabolites included in the SCIEX QT-Meta Method were excluded from the acquisition method because we were primarily interested in microbial consumption of the ZMBI components for this study.

Individual Stock Solutions for each ZMBI media component listed in the **Chemicals, Reagents, and Consumables Section** above were each prepared at solution concentrations of 10 mg/mL in solvent systems described previously[34], and all Stock Solutions were vortex-mixed briefly. Individual Intermediate Solutions were prepared for each ZMBI component at solution concentrations of 100 µg/mL by diluting 10 µL of the respective Stock Solution in 990 µL of methanol: water (1: 1, *v:v*) solution, and all Intermediate Solutions were vortex-mixed briefly. Individual Infusion Solutions were prepared for each ZMBI component at solution concentrations of 500 ng/mL by diluting 5 µL of the respective Intermediate Solution in 995 µL of methanol: water (1: 1, *v:v*) solution, and all Infusion Solutions were vortex-mixed briefly. *Technical Note: if the precursor ion signal was found to be too intense in the prepared infusion solutions (≥7e+7 counts / second in intensity), then the tuning solution was further diluted 5-10-fold directly in the infusion syringe by the addition of an appropriate volume of a neat methanol: water (1: 1, v:v) solution.*

SCIEX QTRAP 7500 System (Quasi-Targeted Metabolomics Method)

The Ultrahigh-Performance Liquid Chromatography (UHPLC)-MS/MS system was comprised of a Shimadzu Nexera 40 Series UHPLC system outfitted with a SIL-30ACMP autosampler (Kyoto, Japan) coupled to a SCIEX QTRAP 7500 system using the SCIEX OS (Ver. 3.3.1.43) software for operational control and to perform relative quantitation.

Metabolite Optimizations on the LC-MS/MS System for the QT-Metabolomics Method

Molecule-specific tuning of the MS system for each of the individual ZMBI components was performed using the MS Method module in SCIEX OS. A volume of ~1 mL of the respective metabolite Infusion Solution was drawn into a 1mL Gastight® [#1001; (Part Number) P/N: 81320] syringe by Hamilton (Reno, Nevada). Each metabolite Infusion Solution was infused into the ionization source of the MS system at a flowrate of 10 µL/min and the mass-to-charge (*m/z*) and precursor ions (i.e., [M+H]+ or [M-H]-) were determined for each metabolite using a Q1 based precursor ion scan with Start mass (Da) and Stop mass (Da) of ± *m/z* 20 bracketing the theoretical precursor ion (*m/z*) for the metabolite being examined. Entrance potential (EP) voltages were not optimized for each metabolite but were set to +15 V or -15 V for positive and negative modes, respectively, for all metabolites. A Q3 Product Ion spectrum was acquired for each metabolite (at the appropriate precursor ion *m/z for each metabolite*) by ramping the collision energy (CE) using the Ramp Compound Parameter Function with Start and Stop CEs of +5 eV to +80 eV for positive mode, or -5 eV and -80 eV for negative mode, respectively, using a 2 eV step size in each instance. Following the Q3 product ion scan, an MRM scan was performed to optimize the CE for the top 4-5 most intense product ions for each metabolite, and the top 2-3 were selected for inclusion in the acquisition method. Collision-cell exit potential (CXP) voltages weren't optimized for each metabolite but rather were set to +15 V or -15 V for positive and negative modes, respectively, for all metabolites.

LC-MS/MS Method for the QT-Meta Method

Chromatographic separations were performed using a Kinetex 2.6-µm F5 (150 × 2.1 mm, 100 Å; cat. no. 00F-4723-AN) analytical column with an attached SecurityGuard F5 (2.1 mm; cat. no. AJO-9322) Ultra Cartridge, both purchased from Phenomenex (Torrance, CA, USA). Chromatographic separation was carried out using mobile phase A (MPA) and mobile phase B (MPB), consisting of 0.1% formic acid in water and 0.1% formic acid in acetonitrile, respectively. The needlewash solution consisted of a mixture of water: methanol: isopropanol: acetonitrile (1: 1: 1: 1*, v: v: v: v*). These solutions were stored sealed at ambient temperature and expired one month after preparation. Operational parameters for the UHPLC system included a mobile phase flowrate of 0.200 mL/min, an autosampler sample bay chilling temperature of +6°C, a column oven heating temperature of +30°C, and a gradient elution program specified as

follows: 0.0-2.1 min, 0% MPB; 2.1-14.0 min, 0-95% MPB; 14.0-16.0 min, 95% MPB; 16.0-16.1 min, 95%-0% MPB; and, 16.1-20.0 min, 0% MPB, with a gradient cycle time of approximately 20.4 minutes per sample.

A High-flow (> 200 μL/min chromatographic flow rate) TurboIonSpray® electrospray ionization (ESI) probe and an E-Lens orthogonal probe were each installed in the OptiFlow Pro Ionization Source that was attached to the inlet of the QTRAP 7500 system. After the MRM optimizations for each of the metabolites were completed, individual injections of 5 μL of aqueous metabolite standards (500 ng/mL) were injected onto the UHPLC-MS/MS system, using the Batch, Queue, and Explore modules in SCIEX OS, in order to empirically determine the retention times (RTs) for each metabolite using the chromatographic system described. The metabolite RTs were used to create a scheduled multiple reaction monitoring (sMRM)-based scanning method that used positive and negative mode polarity switching with Settling and Pause times of 5 ms each, respectively. Ionization source parameters were specified as follows: Ion source gas 1, 30 pounds per square inch (PSI); Ion source gas 2, 50 PSI; Curtain gas, 40 PSI; Collisionally-activated dissociation (CAD) gas, 9 PSI; Source temperature, +350 °C; Positive mode ionspray (IS) voltage, +3,500 V; Negative mode IS voltage: -3,500 V; Apply sMRM triggering, off; and, Q0 dissociation, off.

Triplicate injections of a blank ZMBI culture media were made to create a peak integration method using the Analytics module of SCIEX OS – this method was created specifically to monitor for microbial metabolism of ZMBI components during microbial growth from inoculation to log-phase. Peak integration parameters were optimized for the integration of each metabolite contained in the blank ZMBI culture media in the presence of the other media compounds - this quantitation method file was saved so that it may be used to integrate metabolite peaks present in bacterial-conditioned culture media.
